# Classical Hodgkin lymphoma masquerading as chronic recurrent multifocal osteomyelitis: a case report

**DOI:** 10.1186/s13256-017-1224-4

**Published:** 2017-02-18

**Authors:** Michael Pham, Steven Ressler, Allison Rosenthal, Katalin Kelemen

**Affiliations:** 1 Division of Internal Medicine, Mayo Clinic, Phoenix, AZ USA; 2 Division of Hematology and Oncology, Mayo Clinic, Phoenix, AZ USA; 3 Division of Hematopathology, Mayo Clinic, Phoenix, AZ USA

**Keywords:** Classical Hodgkin lymphoma, Chronic recurrent multifocal osteomyelitis, CRMO, Osseous lymphoma

## Abstract

**Background:**

Hodgkin lymphoma is a hematologic malignancy usually confined to lymphatic structures and commonly associated with constitutional symptoms. Bony involvement and musculoskeletal symptoms are uncommon and typically seen in advanced disease. In this case, we report an unusual presentation of classical Hodgkin lymphoma and highlight diagnostic challenges leading to the misdiagnosis and treatment as chronic recurrent multifocal osteomyelitis.

**Case presentation:**

A 38-year-old white man presented with lower extremity musculoskeletal pain. Imaging studies revealed multifocal lytic and sclerotic osseous axial lesions. Multiple core needle bone marrow and excisional lymph node biopsies were non-diagnostic. Having met the criteria, a tentative diagnosis of chronic recurrent multifocal osteomyelitis was given. He was treated with non-steroidal anti-inflammatory medications with partial clinical response but had persistent symptoms. A second medical opinion was pursued. An open bone marrow biopsy was performed and yielded a diagnosis of classical Hodgkin lymphoma after 13 months of diagnostic uncertainty. A chemotherapy regimen of doxorubicin, bleomycin, vinblastine, and dacarbazine was instituted with complete symptomatic and radiologic response.

**Conclusions:**

This case illustrates diagnostic difficulties of a musculoskeletal presentation of Hodgkin lymphoma, challenges of non-diagnostic bone marrow and lymph node biopsies, and resultant diagnostic delays in delivering a potentially curative therapy. Had the additional open bone marrow biopsy not been performed, the diagnosis and treatment of Hodgkin lymphoma would have been missed.

## Background

Hodgkin lymphoma accounts for approximately 10% of all known lymphomas and has an annual incidence rate of approximately three cases per 100,000 people. Age distribution is classically bimodal and, in industrial nations, peaks at the second and after the sixth decade of life. Treatment regimens developed over the years have become highly effective, and commonly used regimens in use today have remission rates approaching 80 to 90% after the initial course [1].

However, the etiology and mechanism of disease have yet to be fully elucidated. It is thought that the development of B cell-derived neoplastic cells arises from dysregulation of a network of transcription factors at lymphoid germinal and post-germinal centers. On histopathologic examination, pathognomonic Reed–Sternberg cells and other neoplastic variants are present in a mixed inflammatory infiltrate. This infiltration into lymphoid tissue and surrounding organs produces disease and symptoms [2].

In approximately 70% of patients, classical Hodgkin lymphoma presents with painless lymphadenopathy predominantly in the cervical or supraclavicular lymphatic chain distribution and, second most commonly, with an asymptomatic mediastinal mass. In 20 to 40% of patients, classic B-symptoms including fevers, night sweats, or unintentional weight loss are present [3]. Musculoskeletal pain is an uncommon presentation and can sometimes be misdiagnosed as osteomyelitis.

## Case presentation

A previously healthy 38-year-old white man presented with left leg tightness and soreness. He had no significant past medical history but did have an extensive international travel history from his military service. His family history was notable for a maternal history of bladder cancer; a social history disclosed a remote 13-year chewing tobacco history with current cigar smoking accruing to approximately 1.3 cigar-years.

Over a span of approximately 6 months, his left leg tightness and soreness progressed to include his left low back along with sensory changes along his left lateral foot and heel that rendered him with intermittent physical limitation. A review of his symptoms was otherwise unrevealing with no constitutional symptoms. A physical examination was notable for “shotty” supraclavicular lymphadenopathy.

An initial evaluation at an outside institution was ultimately inconclusive. Serial magnetic resonance imaging (MRI) and computed tomography (CT) of his chest, abdomen, and pelvis showed progressive and aggressive-appearing mixed sclerotic-lytic lesions in his iliac and sacral regions. One sacral lesion extended into the left S1 neural foramen. There was associated widespread lymphadenopathy involving the supraclavicular, mediastinal, abdominal, and pelvic regions. Multiple tiny pulmonary nodules up to 10 mm were also present. All imaging appeared consistent with multifocal osteomyelitis although a malignant or infectious process could not be ruled out.

The results of cursory laboratory tests were unremarkable and notable for only a mildly elevated sedimentation rate of 39 mm/hour. Rheumatic serologies and hematologic workup were unrevealing. Extensive infectious studies yielded no identifiable microorganism.

Multiple biopsies were sought. A left axillary lymph node excisional biopsy disclosed reactive lymphadenopathy with scattered pigment-laden macrophages and extensive fatty replacement. Right and left iliac bone marrow biopsies showed marrow fibrosis with focally prominent chronic inflammatory infiltrates. There was no evidence of mycobacterial or fungal organisms. Bone marrow flow cytometry showed no monoclonal B cell population. All biopsies were without evidence of lymphoma or any other neoplastic processes.

A provisional diagnosis of non-bacterial chronic recurrent multifocal osteomyelitis (CRMO) was given. He was treated with opioids, and then transitioned to non-steroidal anti-inflammatory drugs (NSAIDs) and a course of physical therapy. His symptoms improved on this treatment regimen. He received no glucocorticoid during this time.

**Fig. 1:**
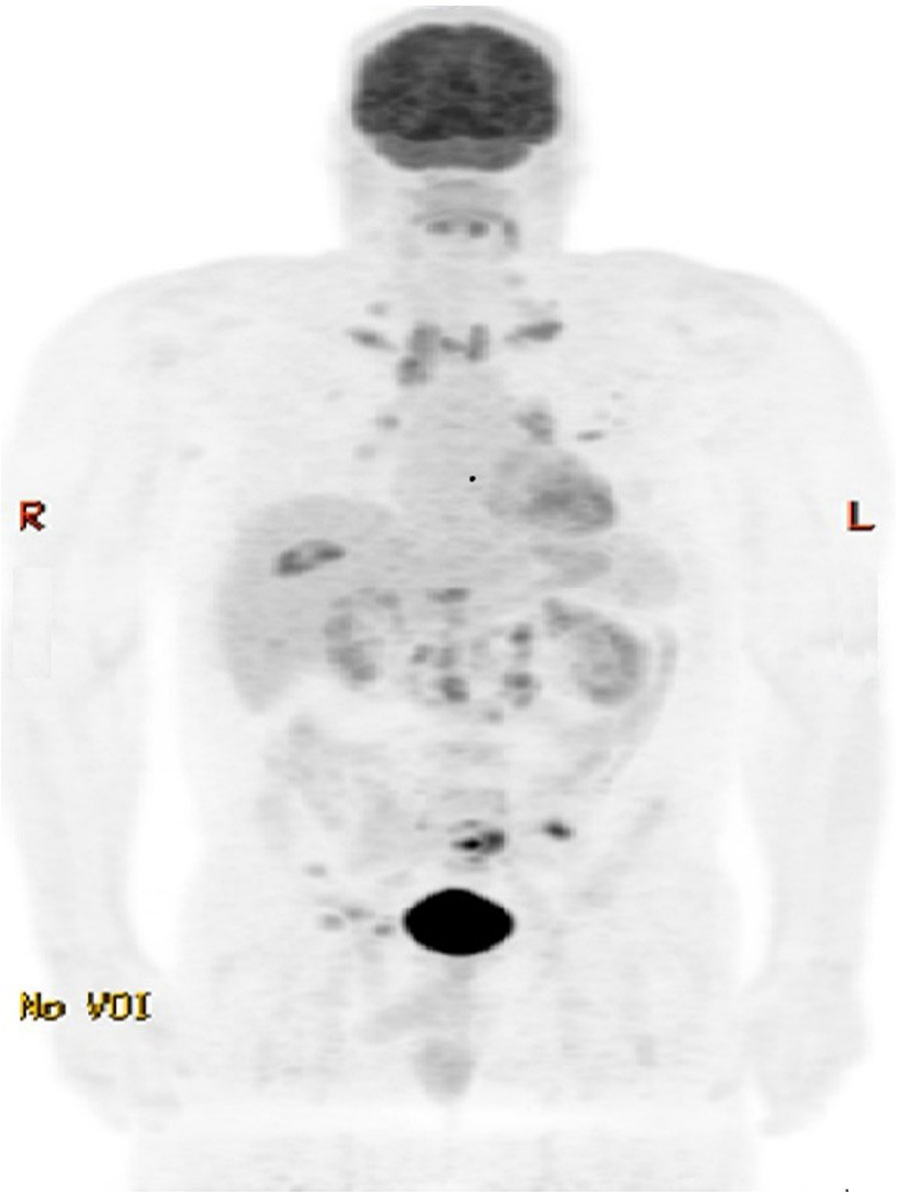
Nuclear positron emission tomography-computed tomography showing multiple foci involving lymph nodes at the paratracheal, left supraclavicular, para-aortic, and right inguinal regions. There was also hypermetabolic activity involving bilateral lung parenchyma and left tenth rib

By the time of presentation to our institution, his clinical course had significantly improved over several months but had not resolved. A repeat positron emission tomography (PET)-CT scan showed hypermetabolic foci throughout his torso involving lymph nodes, bones, and lungs as pictured in Fig. 1. The largest lymph nodes measured up to 1.6×2.0 cm at the left supraclavicular fossa, 2.0 cm at the celiac chain, and 3.1×1.8 cm at the anterior aortocaval chain. Availability of prior study measurements were lacking for comparison. Lytic-sclerotic lesions were re-demonstrated and involved his sacrum, left posterior iliac, right supra-acetabular iliac wing, and left anterior tenth rib. Multiple sub-centimeter lung nodules were re-demonstrated in the upper lobes of his left and right lungs. No splenomegaly was present.

**Fig. 2:**
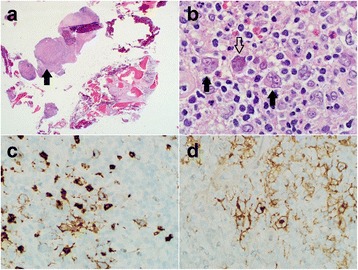
As seen in Panel **a**, low-powered field of the gross bone specimen showing lymphoid tissue nodule (*arrow*) surrounded by faint bands of collagen. Classic multinucleated and bi-lobed Reed–Sternberg cells with surrounding *lacunae* (Panel **b**, *solid arrows*) are shown in a background of a heterogeneous inflammatory infiltrate. A mummified Reed–Sternberg cell is shown (Panel **b**, *open arrow*). Further immunohistochemical staining showed positive cluster of differentiation 15 (Panel **c**) and membrane-distributed cluster of differentiation 30 (Panel **d**) Reed–Sternberg cells characteristic of classical Hodgkin lymphoma

An open bone biopsy of his left iliac crest was then performed and finally revealed a diagnosis of classical Hodgkin lymphoma after 13 months of diagnostic uncertainty. Histologic study demonstrated fibrosis and an inflammatory infiltrate. Newly noted were collections of mummified and lacunar cells with abundant basophilic cytoplasm. Also observed were large multilobed nuclei consistent with Reed–Sternberg and Hodgkin cells as seen in Fig. 2.

He was started on a doxorubicin, bleomycin, vinblastine, and dacarbazine (ABVD) chemotherapy regimen without radiation therapy. Significant clinical and radiologic responses were seen after two cycles. A whole body PET-CT revealed significant reductions in metabolic activity with resolution of his presenting symptoms. After completion of six cycles, the PET-CT was consistent with complete remission.

## Discussion

Here, we report a rare case of classical Hodgkin lymphoma mimicking a presentation of CRMO.

Our patient presented with painless supraclavicular lymphadenopathy and musculoskeletal symptoms which appeared responsive to NSAID therapy. Tobacco use appeared to be the only known exposure risk factor [4]. His laboratory workup was consistent with CRMO and was notable for elevated inflammatory markers, mixed sclerotic-lytic bony lesions on imaging, and multiple core-needle bone marrow and excisional lymph node biopsies showing only a mixed inflammatory infiltrate without evidence of malignancy or infection. At that juncture, he had met and exceeded the diagnostic threshold for CRMO proposed by Jansson *et al*. with three major and three minor criteria met as outlined in Table 1 [5]. Further, the significant and durable response to NSAIDs provided a false positive therapeutic trial as NSAIDs are first-line treatment for CRMO [6, 7]. The pain relief observed may have been multifactorial to decreased bony swelling atop the usual inhibition of cyclooxygenase-derived pain mediators [8, 9].

**Table 1:** Classification of non-bacterial osteitis

Major diagnostic criteria	Minor diagnostic criteria
1. Radiographically proven osteolytic and/or osteosclerotic bone lesion 2. Multifocal bone lesions 3. Pustulosis palmoplantaris or psoriasis 4. Sterile bone biopsy with signs of inflammation and/or fibrosis and sclerosis	A. Normal blood count and good general state of health B. CRP and ESR mildly-to-moderately elevated C. Observation time longer than 6 months D. Hyperostosis E. Associated with other autoimmune diseases apart from pustulosis palmoplantaris or psoriasis F. Grade I or II relatives with autoimmune or auto-inflammatory disease, or with NBO

*CRP* C-reactive protein, *ESR* erythrocyte sedimentation rate, *NBO* non-bacterial osteitis. (2 – As proposed by Jansson *et al*. [5], Classification of Non-Bacterial Osteitis: Retrospective study of clinical, immunological and genetic aspects in 89 patients*. Rheumatology* (2007), 46 (1): 154–160. Permission granted from Oxford University Press.)

Since CRMO is a diagnosis of exclusion, a potential diagnostic pitfall exists in relying on a non-diagnostic bone marrow biopsy to *rule out* a neoplastic cause. This challenge is highlighted with classical Hodgkin lymphoma as diagnostic Reed–Sternberg and Hodgkin cells can be rare and missed due to sampling error.

A survey of the literature reveals sporadic case reports of Hodgkin lymphoma presenting with musculoskeletal pain and an initial misdiagnosis as an osteomyelitis syndrome. Of interest, the vast majority of these cases were associated with primary osseous Hodgkin lymphoma.

Ha-ou-nou *et al*. reported an uncannily similar case of a 35-year-old man presenting with sacral pain and was found to have numerous sacral and iliac osteolytic lesions without B-symptoms or lymphadenopathy [10]. A bone marrow biopsy was non-diagnostic but empiric chemotherapy for Hodgkin lymphoma resulted in resolution of symptoms and lytic lesions [10]. Furthermore, a case series by Ostrowski *et al*. examined 25 patients with biopsy-proven, osseous Hodgkin’s involvement. Nearly all presentations involved bony pain and many had an initial misdiagnosis of osteomyelitis [11]. A handful of other cases with similar presentation and misdiagnosis have been described in the literature [12–15].

It is unclear if our patient had primary osseous classical Hodgkin lymphoma due to an inability to prove the osseous lesions as the primary originating site in the setting of widespread lymphadenopathy already present on his initial medical evaluation. The other possibility would be the more common lymph node-originating Hodgkin lymphoma with progression to bone marrow involvement.

## Conclusions

Ultimately, the presenting complaint of musculoskeletal symptoms in our case affirms the heterogeneity of possible Hodgkin lymphoma presentations and enhances our appreciation for the diagnostic quandary this can entail. The misdiagnosis early in our patient’s clinical course highlights the pitfall of using a non-diagnostic bone marrow and lymph node biopsies to exclude Hodgkin lymphoma. Where tissue biopsy is non-diagnostic, there should be thoughtful consideration for additional tissue biopsies; and, in our case, an open bone biopsy was the critical factor in revealing the diagnosis. Otherwise, a misdiagnosis of CRMO could have easily resulted and delayed therapy in a highly treatable malignancy.

## Abbreviations

ABVD: Adriamycin (doxorubicin), bleomycin, vinblastine, and dacarbazine
CRMO: Chronic recurrent multifocal osteomyelitis
CT: Computed tomography
MRI: Magnetic resonance imaging
NSAID: Non-steroidal anti-inflammatory drug
PET: Positron emission tomography
